# Supplementary material for: Compact Integration of TiO2 Nanoparticles into the Cross-Points of 3D Vertically Stacked Ag Nanowires for Plasmon-Enhanced Photocatalysis
Source: Nanomaterials (Basel). 2019 Mar 20;9(3):468. doi: 10.3390/nano9030468 (PMC6474110; doi:10.3390/nano9030468)
Supplement: Supplementary file 1 [file nanomaterials-09-00468-s001.pdf]

Supplementary Information

Compact Integration of TiO_2 Nanoparticles into the Cross-Points of 3D Vertically Stacked Ag Nanowires for Plasmon-Enhanced Photocatalysis

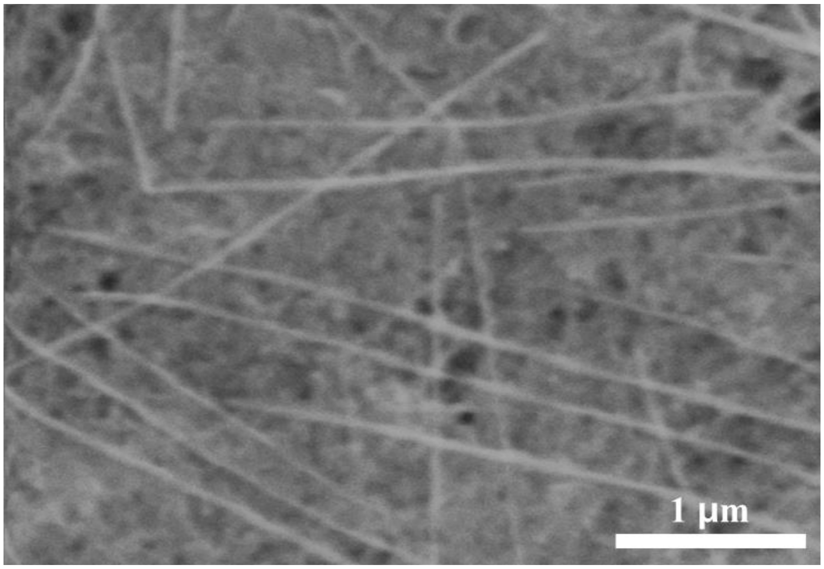

Figure S1. SEM images of 3D hybrid nanostructures upon application of a 0.01 wt% TiO_2 NPs solution onto 3D stacked Ag NWs. Small TiO_2 NPs covered entire pores of the 3D stacked Ag NW substrate.

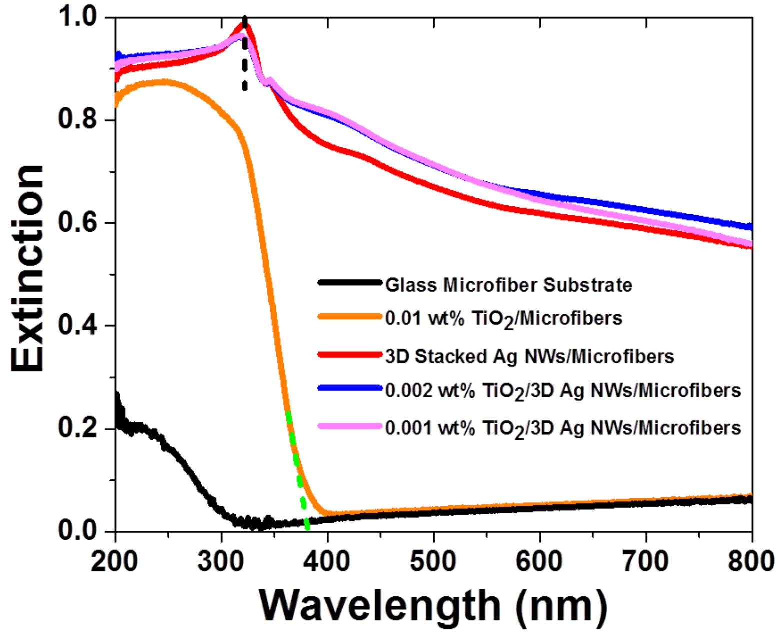

Figure S2. Extinction spectra of different nanomaterials. The black and green dashed lines indicate the interband transitions of Ag and the band gap of TiO_2, respectively.

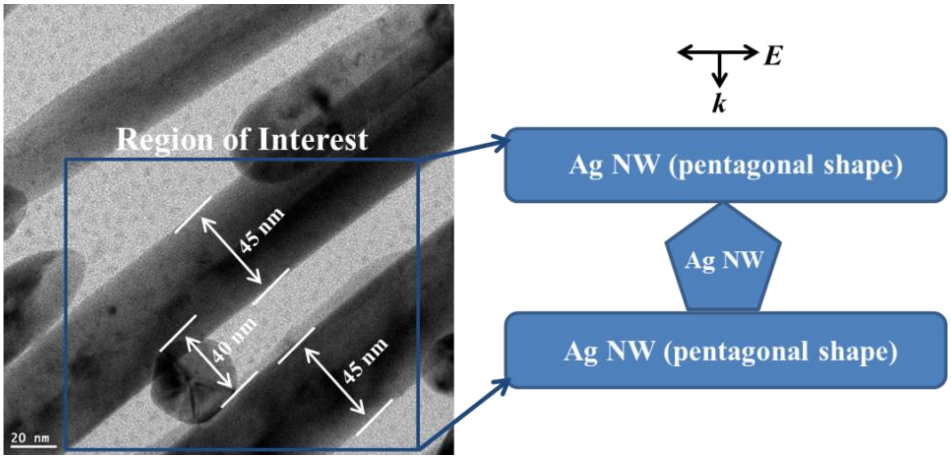

Figure S3. Simulation setup of the 3D stacked Ag NWs. The inset shows a region of interest for a simulation of the cross-points between Ag NWs. The TEM image clearly shows that the Ag NWs were pentagonal in shape.

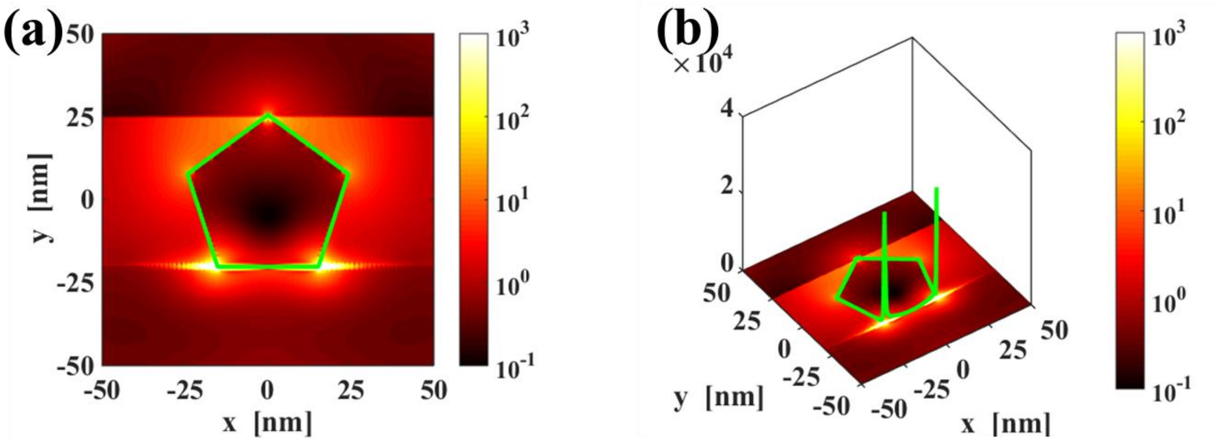

Figure S4. Local electric field intensities at the junctions of crossed Ag NWs. The FDTD method was used in the calculations, along with 450 nm incident. The maximum field enhancement was obtained by searching the maximum values in the domain of the figure. The maximum values were located at the left and right bottom corners of the central pentagon, as shown in (a) and (b). The average field enhancement along the central Ag NW surface was extracted, as indicated by the green lines (0.5 nm away from the Ag NW surface to avoid the staircase effect in the simulation).

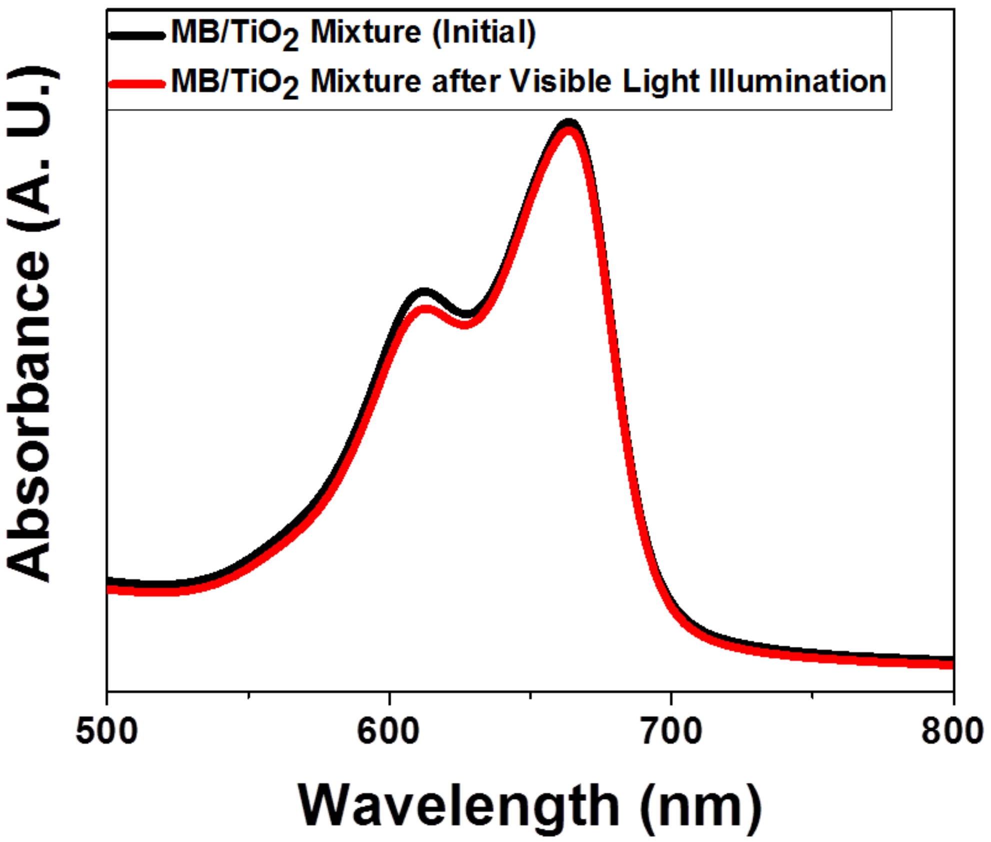

Figure S5. Absorbance changes in the MB solutions after 10 min illumination. 10 mL of 0.05 mM MB aqueous solution were mixed with 4 mL of 0.01 wt% TiO_2 NPs aqueous solution.
